# Routine clinical care data for population pharmacokinetic modeling: the case for Fanhdi/Alphanate in hemophilia A patients

**DOI:** 10.1007/s10928-019-09637-4

**Published:** 2019-05-21

**Authors:** Pierre Chelle, Cindy H. T. Yeung, Santiago Bonanad, Juan Cristóbal Morales Muñoz, Margareth C. Ozelo, Juan Eduardo Megías Vericat, Alfonso Iorio, Jeffrey Spears, Roser Mir, Andrea Edginton

**Affiliations:** 1 School of Pharmacy, University of Waterloo, Waterloo, ON Canada; 2 Department of Health Research Methods, Evidence, and Impact, McMaster University, Hamilton, ON Canada; 3 Hospital Universitari i Politècnic La Fe, Valencia, Spain; 4 Complejo Asistencial Dr. Sótero del Río, Santiago, Chile; 5 Unidade de Hemofilia IHTC ‘Claudio L. P. Correa’, Instituto Nacional de Tecnologia do Sangue, Hemocentro UNICAMP, University of Campinas, Campinas, Brazil; 6 Department of Medicine, McMaster University, Hamilton, ON Canada; 7 Grifols, Research Triangle Park, Durham, NC USA; 8 Grifols, Sant Cugat, Spain

**Keywords:** Hemophilia A, Factor VIII, Population PK, Bayesian forecasting

## Abstract

Fanhdi/Alphanate is a plasma derived factor VIII concentrate used for treating hemophilia A, for which there has not been any dedicated model describing its pharmacokinetics (PK). A population PK model was developed using data extracted from the Web-Accessible Population Pharmacokinetic Service-Hemophilia (WAPPS-Hemo) project. WAPPS-Hemo provided individual PK profiles for hemophilia patients using sparse observations as provided in routine clinical care by hemophilia centers. Plasma factor activity measurements and covariate data from hemophilia A patients on Fanhdi/Alphanate were extracted from the WAPPS-Hemo database. A population PK model was developed using NONMEM and evaluated for suitability for Bayesian forecasting using prediction-corrected visual predictive check (pcVPC), cross validation, limited sampling analysis and external evaluation against a population PK model developed on rich sampling data. Plasma factor activity measurements from 92 patients from 12 centers were used to derive the model. The PK was best described by a 2-compartment model including between subject variability on clearance and central volume, fat free mass as a covariate on clearance, central and peripheral volumes, and age as covariate on clearance. Evaluations showed that the developed population PK model could predict the PK parameters of new individuals based on limited sampling analysis and cross and external evaluations with acceptable precision and bias. This study shows the feasibility of using real-world data for the development of a population PK model. Evaluation and comparison of the model for Bayesian forecasting resulted in similar results as a model developed using rich sampling data.

**Electronic supplementary material:**

The online version of this article (10.1007/s10928-019-09637-4) contains supplementary material, which is available to authorized users.

## Introduction

Fanhdi (Grifols, Barcelona, Spain) and Alphanate (Grifols, Los Angeles, CA, USA) are plasma derived factor VIII (FVIII) concentrates containing von Willebrand factor (VWF) that are used for treating hemophilia A and for which no specific model describing their pharmacokinetics (PK) has been developed. These two concentrates are assumed the same product since they use same manufacturing process [1]. This paper describes the development of a population PK (PopPK) model for Fanhdi/Alphanate using data collected in routine clinical care by hemophilia centers.

Hemophilia A is a genetic bleeding disorder caused by a deficiency in clotting FVIII [2] affecting approximately 1 male in 6500 live births [3]. Patients with severe hemophilia A, defined as an endogenous FVIII activity lower than 0.01 IU/mL, often suffer spontaneous, and recurring joint bleeds, eventually leading to arthropathy. Prophylactic replacement therapy has become the standard treatment for severe hemophilia A. It consists of regular injections of FVIII concentrate and aims at preventing spontaneous joint bleeds by achieving a trough FVIII activity greater than 0.01 IU/mL [4, 5]. As a trough level of 0.01 IU/mL does not prevent all patients from bleeding, higher targets are considered when individualizing treatment [6].

Tailoring a dose and/or a dosing interval to maintain a desired trough in an individual patient is achievable when their individual PK parameters are known. Delineation of the FVIII activity-time profile allows for estimation of relevant PK parameters [7]. Because PK parameters are considerably variable between patients [8], individual PK parameter estimates must be obtained. Such an individual estimation would require 7–10 well distributed blood samples over 72 h, whereas only a more limited number of samples is usually available when studies are performed as part of routine clinical care.

The Web-Accessible Population Pharmacokinetic Service-Hemophilia (WAPPS-Hemo) is a web platform allowing hemophilia care centers to perform PK-tailored dosing [9, 10]. This application estimates individual PK parameters relevant to PK-tailored dosing using limited PK observations and patient information. The approach of WAPPS-Hemo to predict individual PK parameters is Bayesian forecasting using a previously defined PopPK model as prior information.

PopPK models aim to partition the PK variability between subjects (BSV), between occasions (BOV) and within occasion (as remaining residual unexplained error—RUV) as well as the relationship of PK parameters with covariates, such as body weight or age [11]. The principle of Bayesian forecasting is to limit the prediction possibilities of unknown quantities by using prior information on these unknown quantities and their relationship with known quantities [12]. When implementing Bayesian forecasting to PK, PK of new individuals are predicted using the variability of PK and their relationship with covariates as described by PopPK models. Consequently, the better the PK profiles and covariate relationships are described, the more reliable Bayesian forecasting will be. This approach is increasingly used in PK tailoring [13–16] and has been shown to be reliable in a limited sampling environment [16–19].

PopPK models used on the WAPPS-Hemo platform are typically developed using data obtained from a clinical trial, which often are rich sampling data. However, such data are not available for Fanhdi/Alphanate. This work aims to develop a Fanhdi/Alphanate PopPK model using only data collected through WAPPS-Hemo in routine clinical care and evaluate the model’s use for Bayesian forecasting.

## Materials and methods

### Ethical considerations

The WAPPS user agreement allows reuse of the data for modelling and other research purposes, as described in the WAPPS study protocols, approved by the HIREB at McMaster University and registered in clinicaltrial.gov (NCT02061072, NCT03533504).

### Data for model development

Data input into the WAPPS-Hemo platform by clinicians contains individual information relevant for modeling including, but not limited to, dose and duration of infusion; anthropometric data corresponding to body weight (BW), age and height (HT); endogenous (baseline) FVIII activity; measurement assay used (one-stage vs. chromogenic); timing and measured plasma FVIII activity of blood samples.

PK observations from hemophilia A patients receiving an infusion of Fanhdi® or Alphanate® were extracted from the WAPPS-Hemo database on February 16th, 2018. Patients with a history of inhibitors were included, but not those with current inhibitors. Only one occasion per patient was included in the dataset.

HT was not a mandatory covariate in previous versions of WAPPS, and was missing for some patients. When HT was missing, its value was extrapolated from the multilinear regression with BW and age and imputed.

### PopPK model development

The PopPK analysis was performed using non-linear mixed effects modelling as implemented in NONMEM and PDxPop (v7.3 and v5.2, respectively; ICON Development Solutions, Ellicott City, MD, USA). Estimation of the parameters was performed using Laplacian option implemented in NONMEM. Graphical analysis was conducted in MATLAB (R2017b, Mathworks, Natick, MA, USA).

As a first step, observed PK data was assessed as following a 1-, 2- or 3-compartment model following an IV infusion and incorporating any residual FVIII from a previous infusion (predose) and endogenous FVIII activity [9]. Equation 1 provides an example of the time profile activity (*C(t)*) following a 2-compartment model.

1 $$C\left( t \right) = Ae^{ - \alpha t} + Be^{ - \beta t} + endogenous\;FVIII + (predose - endogenous)e^{ - \beta t}$$

Endogenous FVIII was modeled as the value entered by care centers or 0.005 IU/mL when not provided (n = 1 patient only in the evaluation dataset, for which we imputed the value of the most common lower limit of quantification—LLOQ—divided by 2; however LLOQ as low as 0.004 IU/mL are sometimes indicated by centers). Residual FVIII activity was calculated as observed predose activity minus endogenous level. This amount decayed with a rate equal to the terminal decay rate of the compartment model [20].

As the LLOQ is higher than the endogenous factor level in severe hemophilia A patients, samples below LLOQ (BLQ) can be observed. BLQ observations were considered as censored values and handled using the M3 method [21].

Variability in PK parameters (e.g. clearance, volume…) was described as between-subject variability (BSV) using an exponential function [9]. Error on the observations was modeled as residual unexplained variability (RUV) and was tested as additive, proportional and combined error [9].

As a second step, covariate analysis was performed. Covariate relationships were assessed graphically and explored by stepwise forward inclusion (dOFV > 3.84, *p* < 0.05) and backward elimination (dOFV > 6.63, *p* < 0.01) [22]. Body weight (BW), height (HT), age and fat-free mass (FFM) were explored as covariates and normalized to their population median values ($cov_{med}$) to perform the analysis. BW, HT and FFM were tested on each PK parameter (P) using the following equation for any subject *i*:

2 $$TVP_{i} = P_{pop} \left( {\frac{{cov_{i} }}{{cov_{med} }}} \right)^{\theta }$$

where $TVP_{i}$ is the subject PK parameter typical value, $cov_{i}$ his covariate value. $P_{pop}$ represents the PK parameter typical value for the median subject, and $\theta$ a scale factor of the covariate effect on the PK parameter.

The age relationship was modeled as the most significant of linear (Eq. 3) or piecewise linear models (Eq. 4). In the piecewise linear function, the breakpoint was fixed as the median age value of the population, meaning that the typical value was constant for subjects younger than the median age and proportional to age for subjects older than median age (or inversely proportional if $\theta < 0$).

3 $$TVP_{i} = P_{pop} \left( {1 + \theta_{Age} (Age_{i} - Age_{med} )/Age_{med} } \right)$$

4 $$TVP_{i} = P_{pop} \left( {1 + \theta_{Age} max(0, Age_{i} - Age_{med} )/Age_{med} } \right)$$

If two covariates were correlated, only the most significant covariate was kept.

Selection between comparable intermediate models was primarily performed using the objective function value (OFV) and the likelihood ratio test; addition of one parameter to a model was considered significantly better if the OFV decreased by 3.84 or more corresponding to *p* < 0.05 [9]. To complement the selection of the model, diagnostic plots were used to assess the goodness of fit and parameters distributions, especially, the shrinkage of these parameters [23]. If shrinkage of any BSV parameter was higher than 35%, the model was considered over-parameterized and the BSV term was removed. Standard error and confidence intervals on the parameters of selected models were assessed by bootstrap analysis. Bootstrap analysis was performed on 1000 runs by random sampling with replacement accounting for age stratification of the dataset.

### PopPK model evaluation

Prediction-corrected visual predictive check (pcVPC) is a diagnostic tool comparing FVIII activity simulated by the model with observations by plotting percentiles of the observations and simulations vs time [24]. Since the response profile is dependent on dose and covariates, the observations and simulations are normalized by the population predictions of the model allowing a better evaluation of the model. pcVPC was performed by replicating 500 simulations.

Tenfold cross validation was performed to evaluate the ability of the model to predict new data by splitting the data into a learning dataset, used for re-estimating the parameters of the model, and a validation dataset, used for evaluating the model Bayesian predictions. The evaluation consisted in calculating the relative error ($RE_{i}$) of each individual prediction of the new estimated model ($Pred_{i}$—derived from the sub dataset) to the predictions obtained using the original model ($Pred_{i}^{0}$—derived from the complete dataset). For every subject *i* in the evaluation dataset: $RE_{i} = 100\frac{{\left( {Pred_{i} - Pred_{i}^{0} } \right)}}{{Pred_{i}^{0} }}$. The evaluation was repeated 100 times using a random split of the dataset at every iteration. Median and 95th percentile of the absolute value of the relative errors were then computed for clearance (CL) and central volume (V1), as well as for half-life and time spent above a 0.02 IU/mL threshold (TAT2) that were individually derived from the predicted PK parameters. Derivation of TAT2 was obtained by simulating the PK profile using the individual PK parameters, baseline and dose information, then calculating for which time point FVIII was higher than 0.02 IU/mL.

Limited sampling analysis (LSA) evaluates the precision and bias of the model as a function of the number and timing of observations. More specifically, LSA assesses the model robustness in a sparse sampling environment and was performed as described in Brekkan et al. [16]. A virtual dataset was created using the same distribution of demographics and PK as in the final PopPK model. FVIII activity in 1000 virtual subjects receiving 50 IU/kg every Monday-Wednesday-Friday was simulated over 4 weeks. Factor VIII activity from the last Friday dose was used for the analysis. One sample was taken 30 min before and 9 samples were taken after that infusion (at 1, 3, 6, 12, 24, 30, 48, 54, and 72 h). Bayesian predictions of CL and V1, and derived half-life and TAT2, between sparse sampling designs accounting for 2 and 3 observations were compared for precision and bias to the full sampling design.

### External evaluation

New data extracted from WAPPS-Hemo on September 14th, 2018 were used to perform an external evaluation to determine whether the model we derived produced PK outcomes on new patients that were similar to those in the development dataset. Bayesian forecasting was performed to estimate CL, V1, and derive half-life, TAT2 as well as the concentration–time profile for every subject. This evaluation aims to ensure that when the model is used to predict PK profiles in new patients, it does not produce erroneous results.

To assess if this model, built using routine clinical care data, produced similar outcomes as compared to a generic PopPK model for plasma derived FVIII currently used on WAPPS-Hemo [25], Bayesian forecasting was completed with both models for the 49 patients and the outcomes compared by coefficient of determination (R^2^). The generic model was derived using 2760 observations from 310 patients (n = 7 brands) who underwent dense data PK as part of industry and investigator-initiated research projects. Specific covariates are included for plasma derived concentrates accounting for 14 subjects administered with Emoclot and 35 subjects administered with Octanate. This evaluation aims to assess if a plasma-derived FVIII model built using real-world data produces similar outcomes on new patients as compared to a plasma-derived FVIII model built using clinical trial data.

## Results

### Data

**Fig. 1:**
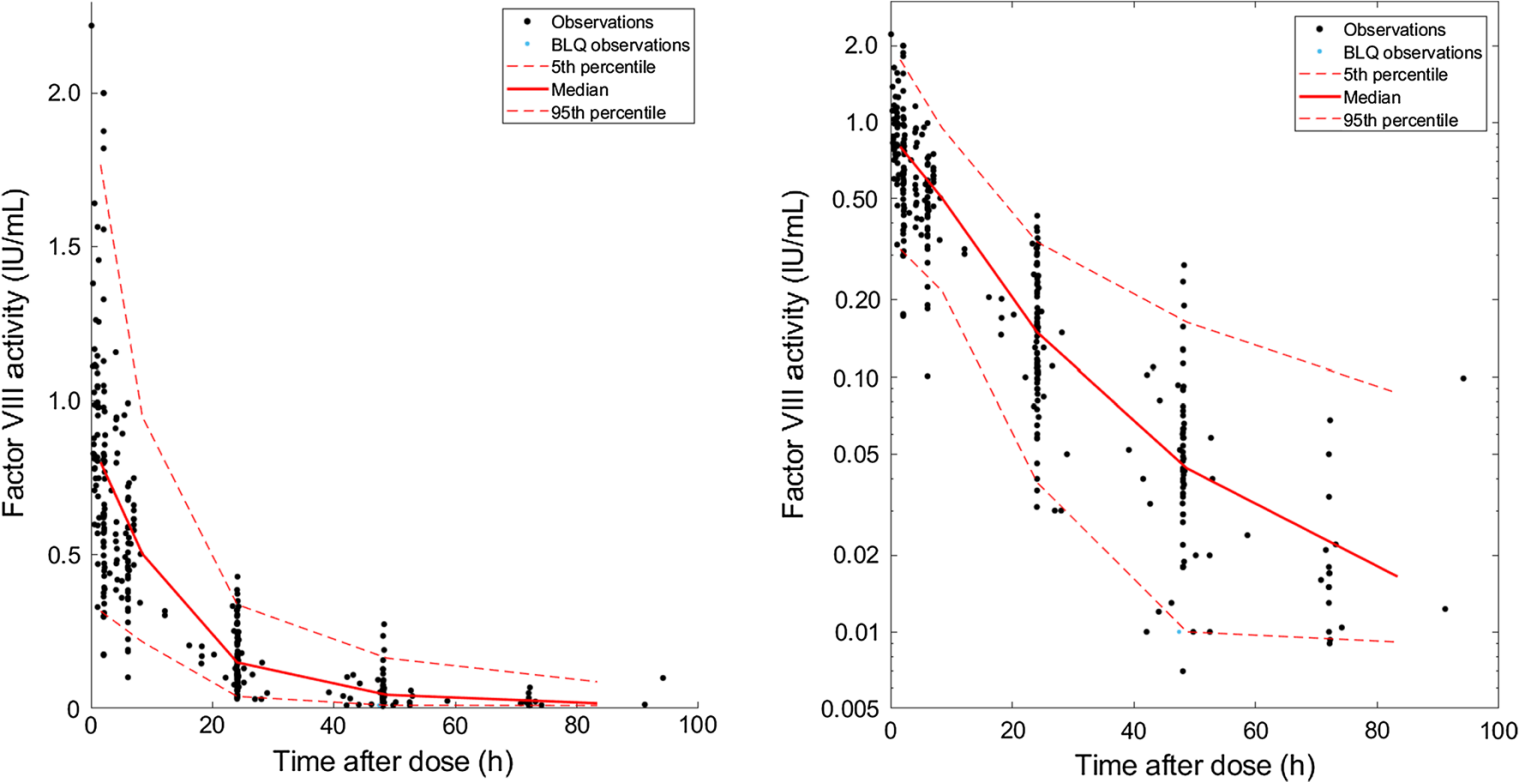
Observations versus time after dose in linear scale (left) and log scale (right)

Ninety-two subjects were included in the development dataset; 67 of which came from 3 centers (Campinas, Brazil; Valencia, Spain; and Santiago, Chile) and the remaining 25 from 9 other centers. These patients were administered one dose of FVIII with between 1 and 8 post-infusion blood samples measured for FVIII activity using the one-stage assay (Table 1). The final dataset contained 386 observations with 13 (3.4%) below LLOQ (BLQ) (LLOQ for these measurements was 0.01 IU/mL). Plots showing the observed FVIII activity versus time following the infusion are shown on Fig. 1.

**Table 1:** Summary of subject demographics for derivation and evaluation populations

	Age (years)	Height (cm)	Body weight (kg)	BMI (kg/m^2^)	Fat free mass (kg)	Sex	Endogenous FVIII level (IU/mL)	Samples/patient
Derivation population								
N	92	87	92	87	87	92 males	92	
Mean	26.1	155.4	59.9	23.4	45.3	–	Severe patients (< 0.01 IU/mL) N = 80 (87.0%)	4.2
SD	18.3	26.6	25.9	5.5	18.0	–		1.5
CV%	70.2%	17.1%	43.3%	23.4%	39.7%	–		35.7%
Median	25	167	63.5	23.9	50.5	–		5
Min	1	73.8	9.68	11.1	7.5	–	< 0.010	1
Max	72	188	119	39.3	73.0	–	0.169	8
Evaluation population								
N	49	49	49	49	49	49 males	48	
Mean	27.1	158.9	59.7	22.2	46.2	–	Severe patients (< 0.01 IU/mL) N = 46 (95.8%)	2.9
SD	17.3	28.9	24.8	5.0	18.0	–		1.3
CV%	63.8%	18.2%	41.6%	22.3%	39.0%	–		46.9%
Median	31	169	65	22.1	53.9	–		3
Min	0.92	76	10.59	13.4	8.1	–	< 0.010	1
Max	60	197	112.5	34.7	73.5	–	0.012	6

### Development of the PopPK model

A 2-compartment model with a proportional error model was selected (dOFV = − 86.1 and + 6.0 compared to 1-, and 3- compartment models respectively). The addition of any additive error did not significantly decrease the OFV. Addition of BSV terms on both CL and V1 led to significant decrease of the OFV (dOFV = − 356.6).

Addition of BSV on Q and/or V2 led to a significant decrease in the OFV, however the shrinkage of these parameters was higher than 44% in each case. Since addition of these parameters was associated with over-parametrization, we maintained the 2-compartment model with BSV on CL and V1 and with proportional RUV for covariate modeling (Intermediate model A).

**Fig. 2:**
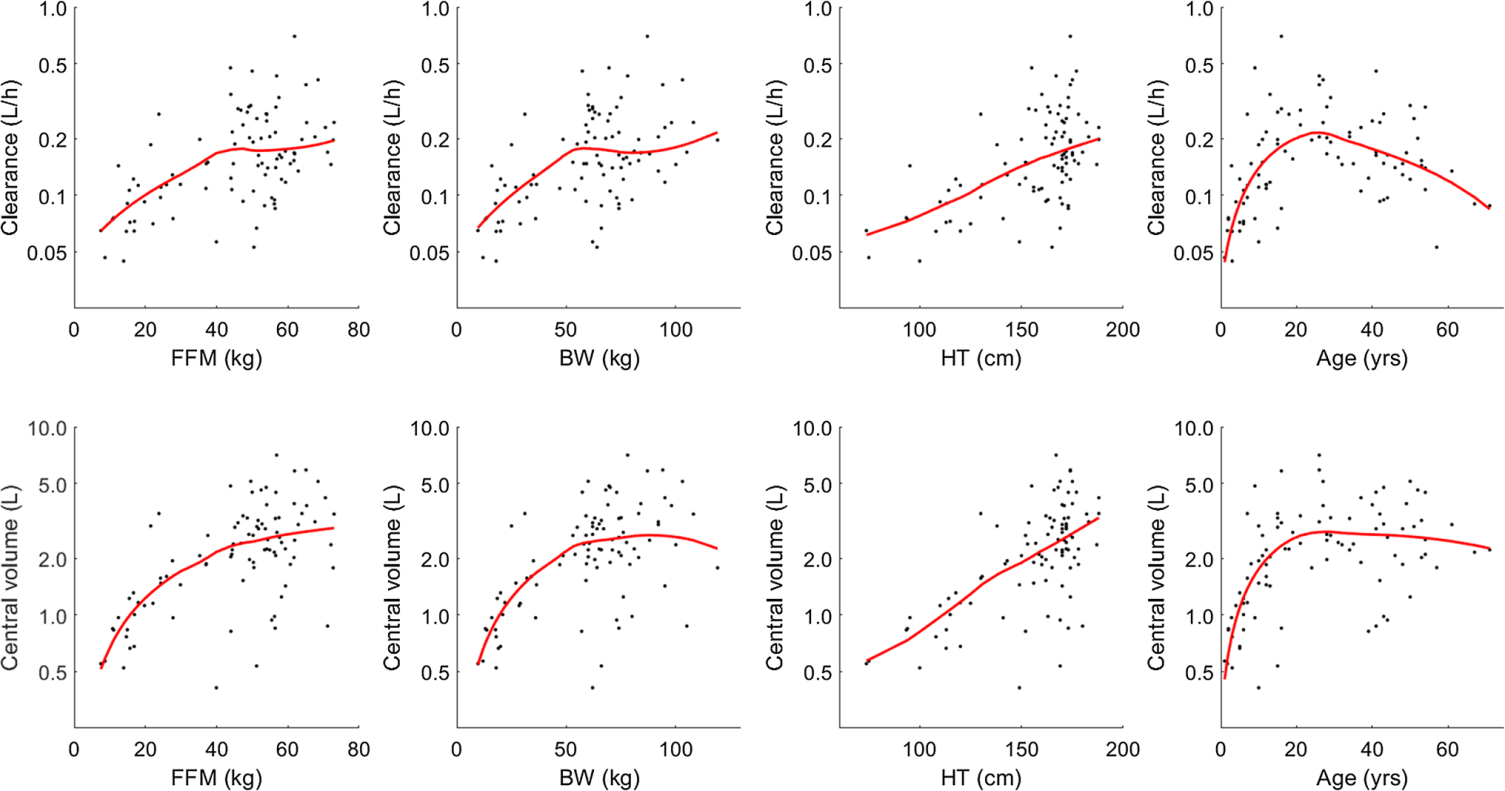
Individual values of CL and V1 versus available covariates

Diagnostic plots (Fig. 2) were produced to assess all the covariates. FFM was the most significant covariate on both CL and V1 with Spearman correlation coefficients 0.41 and 0.49, respectively (dOFV = − 5.0 compared to BW). Addition of this covariate on CL and V1 significantly decreased the OFV (dOFV = − 64.7). Addition of FFM as a covariate on Q and/or V2 was also tested and led to a significant decrease of the OFV when added to V2 (dOFV = − 16.7). Consequently, the FFM effect on CL, V1 and V2 was kept at that stage.

Diagnostic plots (Fig. S1) were produced to assess if there was an age effect on top of FFM. Age was not correlated with individual values of V1 ($\eta_{V1}$). However, age was well correlated with individual values of CL ($\eta_{CL}$). A piecewise linear function best fitted the age effect on CL and this addition significantly decreased the OFV (dOFV = − 13.2) even if it was not significantly better than a linear function in term of OFV decrease (dOFV = − 2.7 compared to linear model). The piecewise function was maintained in the model. Backward elimination did not change the significance of any of the covariates, that were consequently all kept in the model. Finally, diagnostic plots of $\eta_{CL}$ versus $\eta_{V1}$ led to the inclusion of a correlation term between CL and V1 BSV and this effect significantly decreased the OFV (dOFV = − 66.3). The final PopPK model had FFM as a covariate on CL, V1 and V2 as well as Age on CL.

The final PopPK model developed for Fanhdi/Alphanate can be summarized by the following expressions and the values shown in Table 2.

**Table 2:** Population PK model parameters and confidence intervals

Parameter (unit)	Estimate	% RSE	95% CI bootstrap lower bound	95% CI bootstrap upper bound	Definitions RSE: root of standard error CI: confidence interval (obtained from bootstrap)
Structural model					
$CL_{pop}$ (L/h)	0.195	5.69%	0.176	0.217	CL: clearance
$V1_{pop}$ (L)	2.30	7.45%	1.95	2.62	V1: central volume
$Q_{pop}$ (L/h)	0.078	21.3%	0.047	0.120	Q: inter-compartmental clearance
$V2_{pop}$ (L)	0.449	27.1%	0.279	0.776	V2: peripheral volume
Covariate effects					
FFM effect on CL	0.701	12.0%	0.527	0.872	FFM: fat free mass
FFM effect on V1	0.726	13.0%	0.542	0.903	
FFM effect on V2	0.842	72.7%	0.365	3.976	
AGE effect on CL	− 0.302	19.1%	− 0.407	− 0.167	
Between subject variability					
CV of CL	0.456	9.22%	0.365	0.529	CV: coefficient of variation (defined as standard deviation of η)
CV of V1	0.542	11.3%	0.421	0.660	
$Corr_{CL - V1}$	0.797	7.50%	0.669	0.895	Corr: correlation between η
Residual variability					
CV of proportional RUV	0.205	8.23%	0.169	0.232	RUV: residual unexplained variability

5 $$\left\{ {\begin{array}{*{20}c} {CL = CL_{pop} \left( {\frac{FFM}{50.5}} \right)^{{\theta_{FFM - CL} }} \left( {1 + \theta_{AGE - CL} \frac{{{ \hbox{max} }(0, AGE - 25)}}{25}} \right)e^{{\eta_{CL} }} } \\ {\begin{array}{*{20}c} {V1 = V1_{pop} \left( {\frac{FFM}{50.5}} \right)^{{\theta_{FFM - V1} }} e^{{\eta_{V1} }} } \\ {\begin{array}{*{20}c} {Q = Q_{pop} } \\ {V2 = V2_{pop} \left( {\frac{FFM}{50.5}} \right)^{{\theta_{FFM - V2} }} } \\ \end{array} } \\ \end{array} } \\ \end{array} } \right\}$$

With $CL_{pop}$ the typical clearance, $V1_{pop}$ typical central volume, $Q_{pop}$ typical inter-compartment clearance, $V2_{pop}$ typical peripheral volume, $\theta_{FFM}$ FFM effects, $\theta_{AGE - CL}$ age effect on CL, $\eta_{CL}$ BSV term on CL and $\eta_{V1}$ BSV term on V1.

Distributions and correlations of $\eta_{CL}$ and $\eta_{V1}$ were approximatively normally distributed (Fig. S2). Shrinkage of the standard deviations was 4.01% for $\eta_{CL}$ and 9.32% for $\eta_{V1}$ distributions. Compared to the intermediate model A, BSV was reduced by 14% for CL and by 18.3% for V1.

Standard goodness of fit plots for the final Fanhdi/Alphanate PopPK model are provided in Figs. S3 and S4. The observed versus population and individual predicted FVIII activity show that the model described the data well except for high values of FVIII activity, with R^2^ of 0.947 and smoothers of the data lying on the line of unity until 1 IU/mL (Fig. S3). The distribution of CWRES (Fig. S4) was close to a normal distribution and centered around zero along either the population prediction values or the time after dose. RUV shrinkage was 20.8% due to a relatively high amount of sparse sample data (16.3% of the subjects had 2 observations or less).

A comparison was performed on the final model with a different baseline modeling that was assuming a 0.005 IU/mL baseline for every severe patient. Differences between the model estimates were < 1% and the OFV was not significantly modified (dOFV = − 0.7).

### PopPK model evaluations

**Fig. 3:**
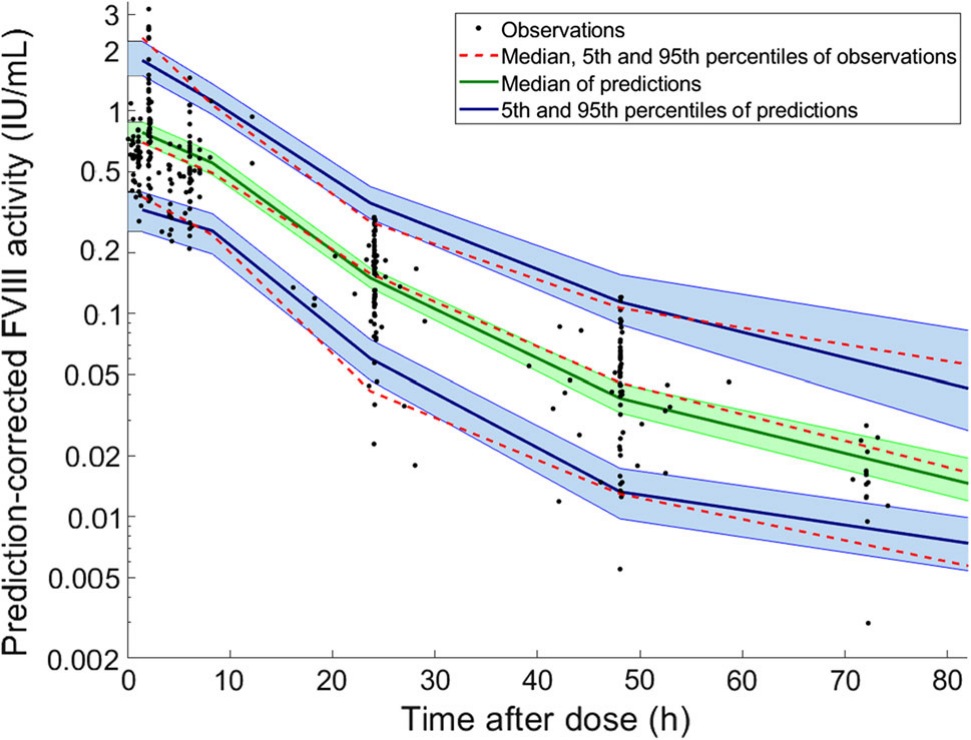
Prediction-corrected visual predictive check of the final model. The dashed lines represent the 5th, 50th, and 95th of the observed data. The solid lines and shaded areas are respectively the corresponding simulated data and their 90% confidence intervals. 500 simulations were performed

Median, 5th and 95th percentiles of observations and predictions are presented on the pcVPC (Fig. 3). Percentiles of observations were within the confidence intervals of predictions in all times.

Tenfold cross validation resulted in using 83 subjects in the learning datasets and 9 subjects in the evaluation datasets. Consequently, 900 Bayesian predictions were compared to the estimates obtained on the complete dataset. The errors obtained on the individual estimates were low, centered around 0 and normally distributed for every parameter. Median—(95th percentile) of absolute errors were 1.01%—(5.27%) for half-life, 0.40%—(3.00%) for TAT2, 0.40%—(2.22%) for CL and 0.67%—(4.76%) for V1.

Mean and coefficient of variation (CV) of CL and V1 predicted using the rich sampling design were similar to the values estimated in the PopPK model (Table S5). Median and 90th percentile of absolute value of relative error between rich and limited sampling designs are summarized in Table S5 for half-life, TAT2, CL and V1.

As expected, for every parameter, the fewer the samples the greater the spread of the error: designs with 2 samples usually led to higher error than designs with 3 samples. Focusing on designs with 3 sample time points median error was lower than 10% for half-life and TAT2. For the same limited sampling designs, the 90th percentile error ranged between 13.0 and 25.2% for half life and between 6.1 and 21.8% for TAT2. Designs that did not have any sampling after 30 h led to higher error on these 2 parameters related to the end phase of the PK profile. Consequently, every 3-sample design tested, except those with only early samples, gave similar results (Fig. S6). Designs assessing the effect of unknown residual FVIII (predose) gave error similar to other designs and no bias (Fig. S6).

**Fig. 4:**
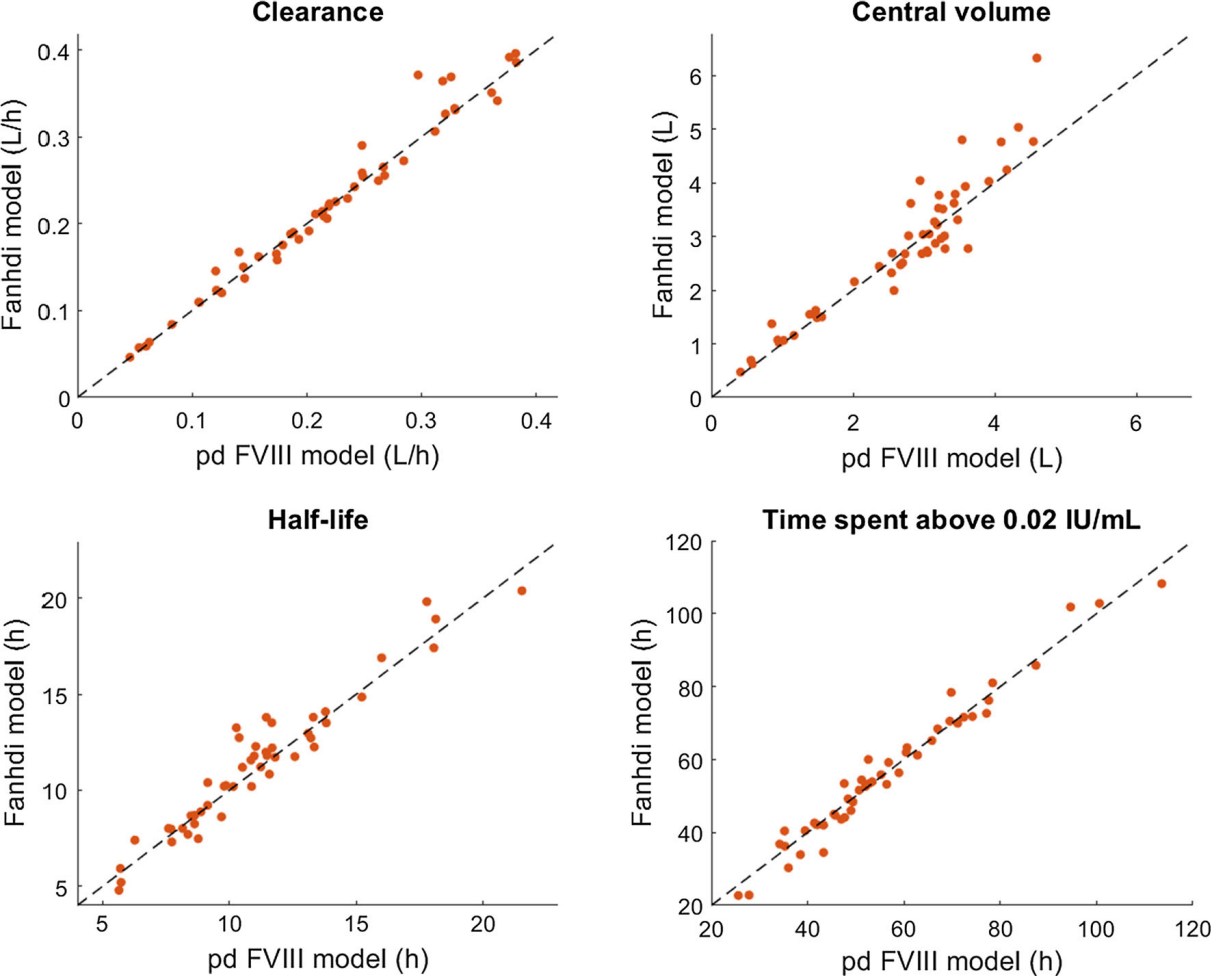
Comparison of CL, V1, half-life and time spent above a 0.02 IU/mL threshold (TAT2) estimated in the evaluation dataset by Fanhdi/Alphanate PopPK model and generic plasma-derived (pd) FVIII model

Forty-nine patients from 13 centers were used for the external evaluation (Table 1). Subjects were ranging from 11 months to 60 years of age and from 10.6 to 112.5 kg. Bayesian forecasting produced half-life and TAT2 estimates similar to the development population. A comparison between Bayesian estimates using the developed Fanhdi/Alphanate model and the generic plasma-derived FVIII model in WAPPS demonstrated a good correlation of CL, V1, half-life and TAT2 estimates (Fig. 4), with coefficients of determination (R^2^) for equality respectively equal to 0.97, 0.84, 0.91 and 0.96. V1 was less correlated, however, the differences were found on patients who did not have any observation of peak activity.

## Discussion/conclusion

This study describes the development and evaluation of a PopPK model built from routine clinical data that were input into the WAPPS-Hemo platform. The purpose of this PopPK model is for use as a prior for Bayesian forecasting in the WAPPS-Hemo platform.

FFM was a better predictor of clearance and volume as compared to BW. From a physiological point of view this is expected considering FVIII is a macro-molecule and has very limited distribution confined to vascular space and some interstitial. Previous PopPK models of FVIII used an allometric exponent on BW to account for a mismatch with overweight patients [26]. In more recent modeling, lean body weight has replaced total body weight as a covariate [27]. Lean body weight and FFM are similar in concept but the equation used to generate FFM [22] used a high quality method (dual energy x-ray absorptiometry) across the entire age spectrum (3–82 years) for which is was developed and therefore provides confidence that FFM was appropriately assessed for patients used to build the Fanhdi/Alphanate model. Despite not being precisely estimated (RSE of 72.7%), FFM was kept on peripheral volume because, from a physiological stand-point, smaller patients with low FFM and low central volume, are more likely to have a smaller peripheral volume.

Based on a mechanistic understanding of clearance for FVIII, vWF levels should be the most relevant covariate on CL [28], but the data was not available for many patients. As a result, we used age as a proxy of vWF as they are correlated [28]. The primary reason for using a piecewise linear model for age was to prevent any interference between correlated covariates. In children and teenagers, age and FFM are strongly correlated. An age effect on CL in children would have led to an over-estimation of FFM on CL. This can lead to a non-physiological artifact effect, such as half-life decreasing when FFM increases. Blood type was also not available on many patients, otherwise it would have been tested as an additional covariate to supplement age as a vWF surrogate [28].

The analysis used to develop this PopPK model is similar to what is already performed for other FVIII concentrates [17, 26, 27, 29–33]. Table 3 summarizes some features of PopPK models available in the literature. Our analysis for Fanhdi/Alphanate led to similar outcomes in terms of model structure with a 2-compartment model best describing the PK profile; as well as covariates of age for CL and body size for V1 and CL. Typical values for PK parameters as well as proportional error were within the same range as other FVIII PopPK models; however, BSV for CL and V1 were higher in this PopPK model. This is likely due to both the sparsity of some data along with diversity in measurement standards and methods between hemophilia centers. Inter-laboratory variability in activity measurement is usually higher than 10% and was reported up to 35% for the one-stage assay [34, 35]. Since data were entered by 12 different hemophilia centers worldwide for the development data, this variability may be captured in CL and V1 BSV.

**Table 3:** Summary of FVIII PopPK models available in literature

References	FVIII concentrate	Number of subjects	Age (years) Median (range)	BW (kg) Median (range)	CL TV (L/h)—BSV (CV) covariates	V1 TV (L)—BSV (CV) covariates	Q TV (L/h)	V2 TV (L)	RUV P (CV) A (SD in IU/mL)
	Fanhdi/Alphanate	92	25 (1–72)	63.5 (9.7–119)	0.195^a^—45.6% FFM, Age	2.30^a^—54.2% FFM	0.078	0.449	P: 20.5%
Abrantes [29]	Refacto/ Xyntha	754	23 (0.003–73)	69 (3–134)	0.276^b^—30.5% inhibitors, age, study	2.45^b^—0% BW	2.51	0.923	P: 19.2%
Garmann [27]	Kovaltry	183	22 (1–61)	60 (11–124)	0.188^c^—37% LBW	3.00^c^—11.2% LBW	0.190	0.637	P: 26.7% A: 0.011
Shah [36]	Kovaltry (joint with Advate)	18	36 (19–64)	80 (55–99)	0.151—27.2%	2.36—7.93%	0.159	0.535	P: 5.73%
Zhang [30]	Afstyla	106	23 (1–60)	60.8 (10–106)	0.212^d^—24.1% BW, VWF	3.36^d^—19.7% BW	0.134	0.265	P: 10.9% A: 0.011
Bjorkman [26]	Advate	152	22 (1.1–66)	56 (11–108)	0.193^e^—30% BW, age	2.22^e^- 21% BW	0.147	0.73	A: 0.089
Bolon-Larger [17]	Multiple plasma derived and recombinant	51	39.5 (7–77)	68 (21–120)	0.177^f^—45.4%	2.82^f^—21.1% BSA	0.152	1.54	Not specified
Hazendonk [31]	Multiple plasma derived and recombinant	119	40 (0.2–78)	75 (5–111)	0.160^g^—36% BW, age, Blood group O, surgery	2.81^g^—26% BW, age	0.170	1.89	P: 18-23% A: 0.05-0.14
Nestorov [32]	rFVIII-Fc Advate	180 118	30 30	73 73	0.173 ^h^—25.1% VWF 0.253 ^h^—30.4%	3.68 ^h^—13.4% BW, HCT 3.46 ^h^—16.2% BW	0.0279 0.0548	0.409 0.494	P: 15.4% A: 0.0024 P:16.8% A: 0.0011
Karafoulidou [33]	Refacto	28	34 (18–70)	75 (54–104)	0.393^i^—38.9% BW	4.86^i^—13.0% BW, viral status	_^l^	_	15.2%
Jimenez [37]	Novo8	76	20 (1–60)	75 (12–107)	0.302^j^—32.0% BW, age	3.46^j^—22.0% BW	_	_	Not specified
McEneny-King [25]	Multiple plasma derived and recombinant	400	22.5 (1–67)	67.1 (10.6–140)	0.275^k^—40.9% FFM, age, concentrate	3.18^k^—30.7% FFM, concentrate	0.153	0.559	P: 16.2% A: 0.0095

*TV* typical value, *BSV* between subject variability, *P* proportional, *A* additive, *LBW* lean body weight, *VWF* Von Willebrand factor, *BSA* body surface area, *HCT* hematocrit

^a^Typical value for a 25 year old—50.5 kg FFM subject

^b^Typical value for a 20 year old—70 kg BW subject

^c^Typical value for a 51.1 kg LBW subject

^d^Typical value for a 113% VWF activity—68 kg BW subject

^e^Typical value for a 22 year old—56 kg BW subject

^f^Typical value for a 1.80 m^2^ BSA and 68 kg BW subject

^g^Typical value for a 20 year old—68 kg BW subject

^h^Typical value for a 45 of HCT—118 VWF—73 kg BW subject

^i^Typical value for a 75 kg BW subject

^j^Typical value for a 20 year old—75 kg BW subject

^k^Typical value for a 22 year old—53 kg FFM subject

^l^Was used to indicate a 1 compartment model (in which neither Q nor V2 are defined)

Since the purpose of this PopPK model is to be used as a prior model for Bayesian forecasting on the WAPPS-Hemo platform, we focused on dedicated evaluation methods and criteria. Half-life and time spent above a certain activity threshold are two major criteria when designing a prophylactic treatment regimen. pcVPC showed that the PopPK is able to simulate the observed data. Tenfold cross validation showed that new data can be predicted by Bayesian forecasting with sufficient precision and no bias.

As suggested by Björkman et al. [18], combining a PopPK model with limited sampling strategies can be useful for the prediction of FVIII PK. Indeed, LSA provides relevant information on the reliability of the PopPK model when predicting PK parameters from sparse data as well as where this sparse sample data is informative to the prediction. In our analysis, half-life and TAT2 are parameters better predicted using late observations where using observations before 30 h alone does not bring individual information to these parameters. In terms of reliability, no bias was observed in the predictions; errors on CL and V1 are acceptable considering that BSV had a 50% CV for both parameters. Theoretically, since our LSA was performed on simulated data, we cannot exclude that a similar analysis performed on densely sampled data would provide different results. However, the LSA results are in line with similar analyses performed on dense data for other factor VIII concentrates, and can guide practice until eventually confirmed or replaced by new data.

External evaluation with comparison to a generic plasma-derived FVIII PopPK model developed using clinical trial data added further value to this newly developed PopPK model. First, the agreement between these two different models in term of FVIII activity-time profile and PK estimates were good. Differences between the predictions of these two models were only found in cases where the observations did not bring individual information to the predicted parameter, and the model prediction consequently reflected the population value for the subject. This was especially the case for V1, where discrepancies of predictions corresponded to patients having samples observed after 24 h. With respect to the assessment of Bayesian forecasting using new data from WAPPS, the aim was to check that the PopPK model produced reasonable Bayesian predictions in an external cohort before being released to the Web Service. Since the true PK profiles and parameters are not known for the external cohort, the comparison with the derivation data is limited to checking that the new predictions are in reasonable agreement with the derivation data. It is however understood that the Bayesian predictions may differ between cohorts even given the same covariates.

The work described in this paper shows the feasibility of developing a PopPK model from routine clinical data and using it for Bayesian analysis. This PopPK model was comparable with PopPK models describing clinical trial data. It encompasses a wide range of age and body weight allowing a relevant description of the covariate effects. Consequently, the model and its inputs might be more suited to predict new clinical routine data that can’t be captured by clinical trials.

Since optional information can be input in the platform, further analysis, especially in terms of covariates, can be explored with such data. From this analysis, a promising perspective would be to supplement clinical trial data with routine practice data in order to build future PopPK models. On one hand, dense data from clinical trials brings stability and a good description of the shape of the PK curves; on the other hand, sparse data from routine practice widens the possible observations, input and covariates of the model.

## Electronic supplementary material

Below is the link to the electronic supplementary material.

Supplementary material 1 (DOCX 869 kb)
