# Correction to: Routine clinical care data for population pharmacokinetic modeling: the case for Fanhdi/Alphanate in hemophilia A patients

**DOI:** 10.1007/s10928-019-09647-2

**Published:** 2019-07-13

**Authors:** Pierre Chelle, Cindy H. T. Yeung, Santiago Bonanad, Juan Cristóbal Morales Muñoz, Margareth C. Ozelo, Juan Eduardo Megías Vericat, Alfonso Iorio, Jeffrey Spears, Roser Mir, Andrea Edginton

**Affiliations:** 1 School of Pharmacy, University of Waterloo, Waterloo, ON Canada; 2 Department of Health Research Methods, Evidence, and Impact, McMaster University, Hamilton, ON Canada; 3 Hospital Universitari i Politècnic La Fe, Valencia, Spain; 4 Complejo Asistencial Dr. Sótero del Río, Santiago, Chile; 5 Unidade de Hemofilia IHTC ‘Claudio L. P. Correa’, Instituto Nacional de Tecnologia do Sangue, Hemocentro UNICAMP, University of Campinas, Campinas, Brazil; 6 Department of Medicine, McMaster University, Hamilton, ON Canada; 7 Grifols, Research Triangle Park, Durham, NC USA; 8 Grifols, Sant Cugat, Spain

## Correction to: Journal of Pharmacokinetics and Pharmacodynamics 10.1007/s10928-019-09637-4

The article Routine clinical care data for population pharmacokinetic modeling: the case for Fanhdi/Alphanate in hemophilia A patients, written by Pierre Chelle, Cindy H. T. Yeung, Santiago Bonanad, Juan Cristóbal Morales Muñoz, Margareth C. Ozelo, Juan Eduardo Megías Vericat, Alfonso Iorio, Jeffrey Spears, Roser Mir, Andrea Edginton, was originally published electronically on the publisher's internet portal (currently SpringerLink) on 21 May 2019 without open access.

With the author(s)' decision to opt for Open Choice the copyright of the article changed on July 2019 to © The Author(s) 2019 and the article is forthwith distributed under the terms of the Creative Commons Attribution 4.0 International License (http://creativecommons.org/licenses/by/4.0/), which permits use, duplication, adaptation, distribution and reproduction in any medium or format, as long as you give appropriate credit to the original author(s) and the source, provide a link to the Creative Commons license and indicate if changes were made.

