# Supplementary material for: Routine clinical care data for population pharmacokinetic modeling: the case for Fanhdi/Alphanate in hemophilia A patients
Source: J Pharmacokinet Pharmacodyn. 2019 May 21;46(5):427–38. doi: 10.1007/s10928-019-09637-4 (PMC6820598; doi:10.1007/s10928-019-09637-4)
Supplement: Supplementary file 1 — Supplementary material 1 (DOCX 869 kb) [file 10928_2019_9637_MOESM1_ESM.docx]

**Supplementary Files**

The figures shown in supplementary files correspond to diagnostic plots and evaluations realized after having developed the final population PK model for Fanhdi/Alphanate.

Figure S1: Individual deviations from typical subjects (η) of CL and V1 vs available covariates after addition of FFM on CL, V1, V2.

Figure S2: Distribution and relationship described between BSV terms of the PopPK model. Top left and bottom right: histograms of individual values of CL and V1 (𝜂), blue corresponds to empirical distribution, red is assumed normal distribution. Top right and bottom left: scatter plots of individual values of CL vs V1

Figure S3: Goodness of fit plots on a linear (left) and log scale (right). Red crosses correspond to individual predictions, blue crosses to population predictions, and small dots to prediction associated to BLQ observations.

Figure S4: Conditional weighted residuals (CWRES) plots. Top left: QQ plot. Top right: histogram of CWRES distribution. Bottom left: plot of CWRES vs population predictions. Bottom right: plot of CWRES vs time after dose.

Table S5: Results of limited sampling analysis: median and 90^th^ percentile error between predictions of rich and limited sampling designs.

Figure S6: Limited analysis sampling results: boxplot of the error on half-life predictions for each 2- and 3- samples designs tested.


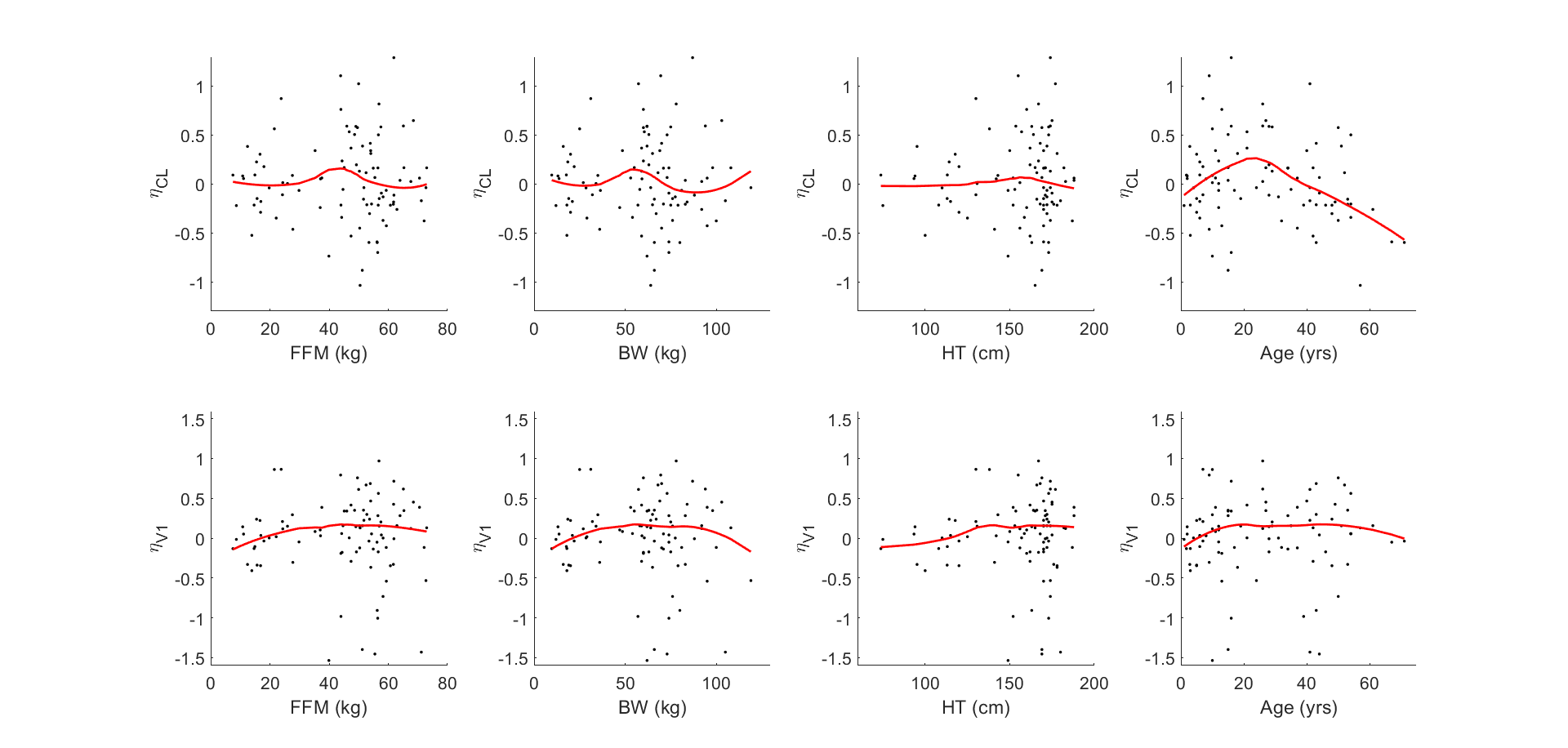


Figure S1: Individual deviations from typical subjects (η) of CL and V1 vs available covariates after addition of FFM on Cl, V1, V2.


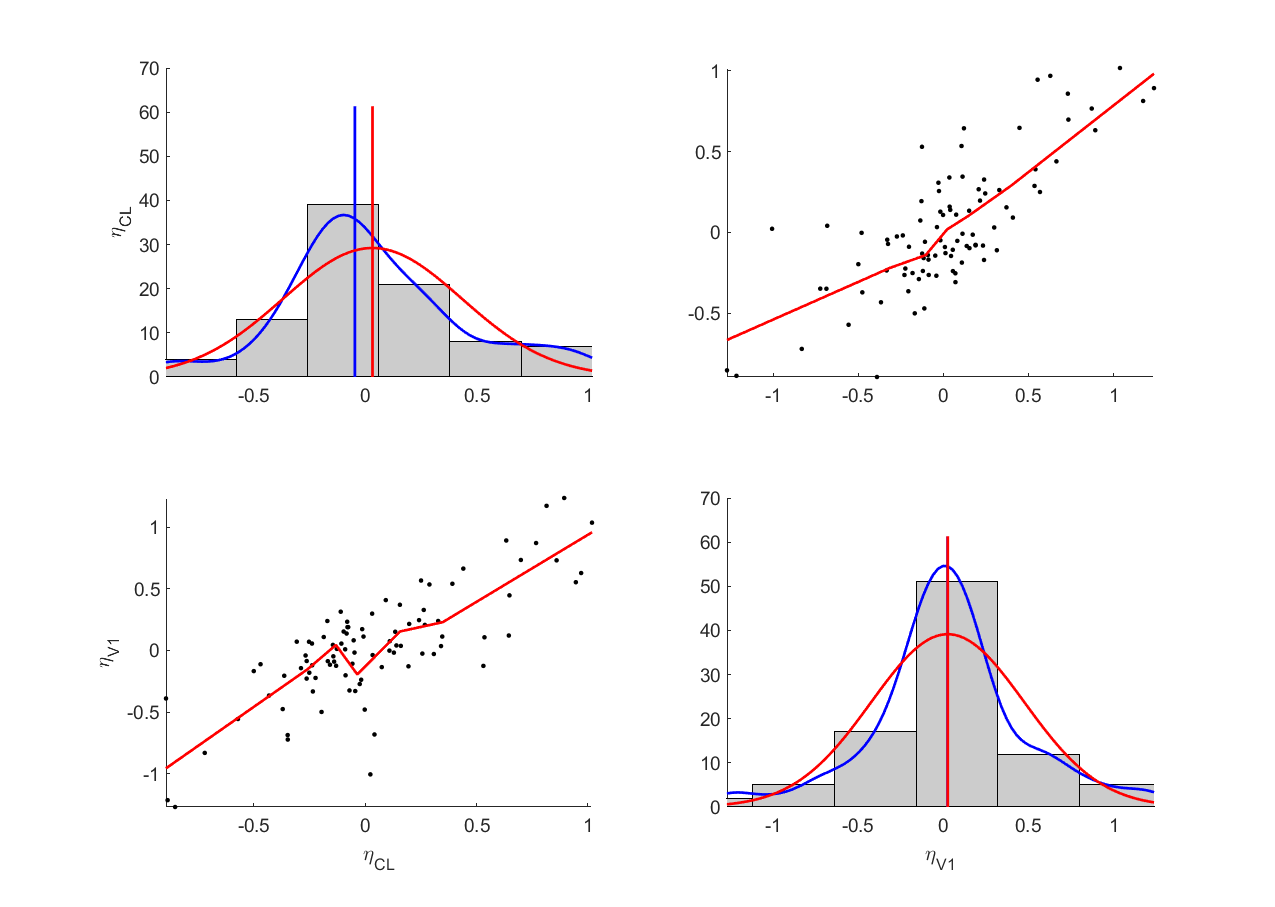


Figure S2: Distribution and relationship described between BSV terms of the PopPK model. Top left and bottom right: histograms of individual values of CL and V1 (𝜂), blue corresponds to empirical distribution, red is assumed normal distribution. Top right and bottom left: scatter plots of individual values of CL vs V1


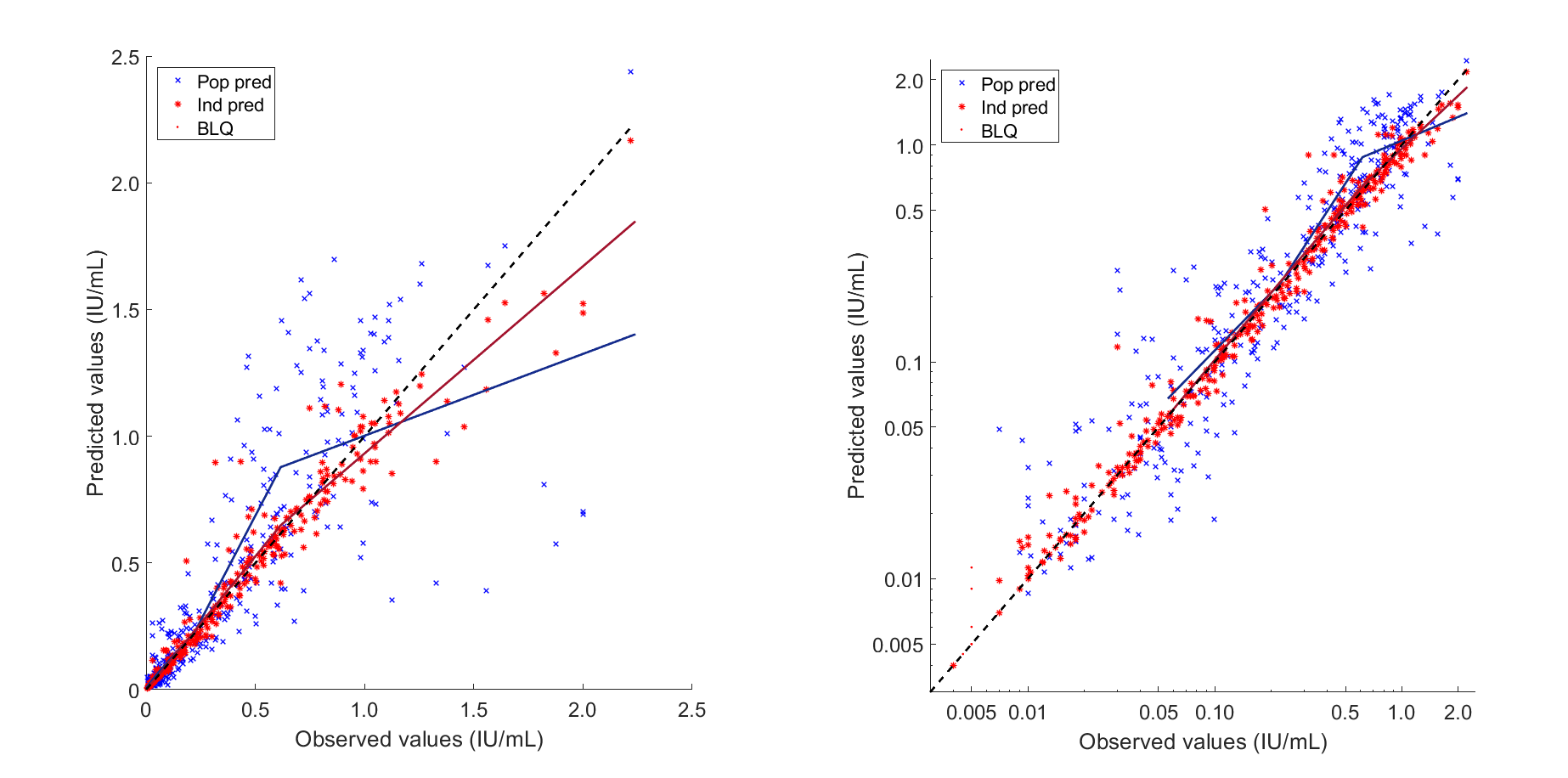


Figure S3: Goodness of fit plots on a linear (left) and log scale (right). Red crosses correspond to individual predictions, blue crosses to population predictions, and small dots to prediction associated to BLQ observations.


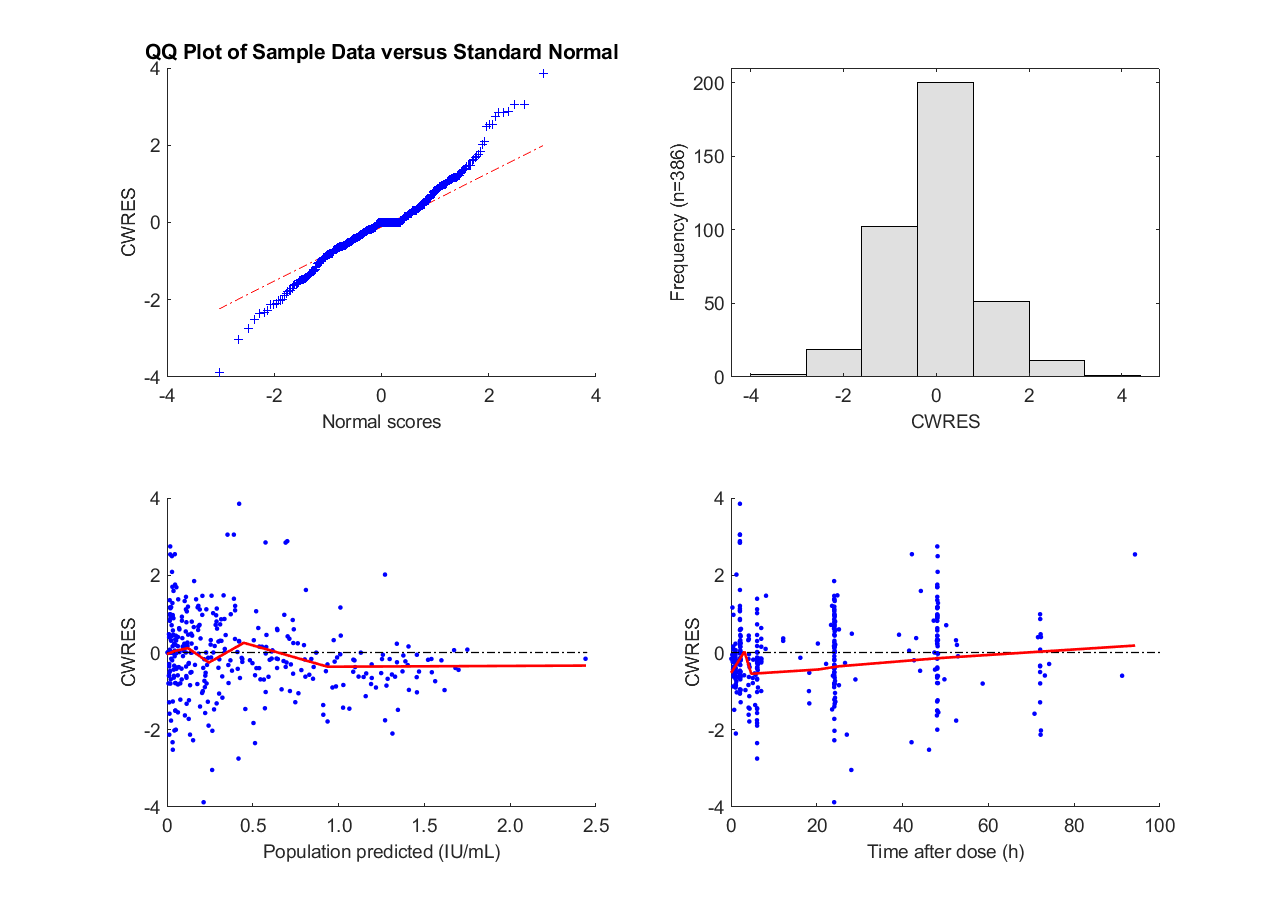


Figure S4: Conditional weighted residuals (CWRES) plots. Top left: QQ plot. Top right: histogram of CWRES distribution. Bottom left: plot of CWRES vs population predictions. Bottom right: plot of CWRES vs time after dose.

Table S5: Results of limited sampling analysis: median and 90^th^ percentile error between predictions of rich and limited sampling designs.

| **Description**  **(sampling time points)** | **Sampling  ratio (%)** | **Half-life**  **Median – 90^th^ percentile error** | **CL**  **Median – 90^th^ percentile error** | **V1**  **Median – 90^th^ percentile error** | **TAT2**  **Median – 90^th^ percentile error** |
| --- | --- | --- | --- | --- | --- |
| pre-1-3-6-12- 24-30-48-54-72h | 100 % | Mean: 12.6h –  CV: 33.7% | Mean: 0.18L/h - CV: 59.2% | Mean: 2.41L –  CV: 65.9% | Mean: 72.1h –  CV: 39.6% |
| pre-1-24h | 30 % | 8.58 % - 22.1 % | 9.19 % - 25.4 % | 10.7 % - 27.9 % | 8.27 % - 21.2 % |
| pre-1-30h | 30 % | 7.07 % - 18.4 % | 8.20 % - 24.0 % | 10.5 % - 28.1 % | 6.64 % - 17.3 % |
| pre-1-48h | 30 % | 4.57 % - 13.0 % | 7.70 % - 22.1 % | 10.8 % - 30.0 % | 4.58 % - 12.0 % |
| pre-1-54h | 30 % | 4.67 % - 13.2 % | 8.48 % - 22.4 % | 10.6 % - 30.0 % | 4.44 % - 11.6 % |
| pre-1-72h | 30 % | 4.28 % - 15.1 % | 9.26 % - 25.4 % | 10.7 % - 30.6 % | 4.41 % - 16.6 % |
| pre-3-24h | 30 % | 9.51 % - 25.2 % | 9.28 % - 26.8 % | 13.1 % - 35.9 % | 8.81 % - 21.8 % |
| pre-3-30h | 30 % | 7.51 % - 20.5 % | 8.81 % - 25.7 % | 12.9 % - 38.0 % | 6.70 % - 18.0 % |
| pre-3-48h | 30 % | 4.75 % - 13.6 % | 8.86 % - 25.3 % | 12.9 % - 37.4 % | 4.66 % - 12.5 % |
| pre-3-54h | 30 % | 4.94 % - 13.5 % | 9.33 % - 24.8 % | 12.9 % - 38.9 % | 4.45 % - 11.8 % |
| pre-3-72h | 30 % | 4.64 % - 15.2 % | 9.44 % - 26.1 % | 12.9 % - 39.1 % | 4.38 % - 16.3 % |
| 24-30-48h | 30 % | 7.12 % - 19.4 % | 14.3 % - 32.8 % | 25.2 % - 60.2 % | 3.93 % - 11.5 % |
| 24-30-54h | 30 % | 7.03 % - 18.2 % | 14.6 % - 32.2 % | 25.0 % - 61.0 % | 3.59 % - 10.3 % |
| 24-30-72h | 30 % | 5.92 % - 15.2 % | 13.9 % - 31.7 % | 24.2 % - 55.9 % | 3.10 % - 7.86 % |
| 24-48-54h | 30 % | 6.11 % - 16.4 % | 15.2 % - 35.0 % | 27.3 % - 65.4 % | 2.59 % - 8.84 % |
| 24-48-72h | 30 % | 5.69 % - 14.0 % | 15.4 % - 34.0 % | 25.3 % - 61.7 % | 2.63 % - 6.98 % |
| 30-48-54h | 30 % | 6.51 % - 17.4 % | 15.1 % - 35.8 % | 27.4 % - 66.0 % | 2.47 % - 8.75 % |
| 30-48-72h | 30 % | 5.41 % - 14.6 % | 15.3 % - 35.2 % | 25.4 % - 61.1 % | 2.59 % - 6.64 % |
| 30-54-72h | 30 % | 5.43 % - 14.5 % | 14.7 % - 35.6 % | 25.3 % - 61.0 % | 2.27 % - 6.55 % |
| 48-54-72h | 30 % | 5.72 % - 15.3 % | 18.4 % - 41.7 % | 29.6 % - 70.4 % | 1.87 % - 6.09 % |
| 24-30h | 20 % | 10.3 % - 24.4 % | 14.5 % - 32.8 % | 29.2 % - 67.2 % | 6.51 % - 18.2 % |
| 24-48h | 20 % | 7.21 % - 20.0 % | 15.3 % - 35.0 % | 27.3 % - 65.7 % | 4.51 % - 12.4 % |
| 24-54h | 20 % | 7.09 % - 18.6 % | 15.3 % - 35.8 % | 27.4 % - 67.1 % | 4.11 % - 11.1 % |
| 24-72h | 20 % | 6.23 % - 16.1 % | 15.7 % - 37.2 % | 26.3 % - 65.6 % | 3.74 % - 10.3 % |
| 30-48h | 20 % | 7.48 % - 19.6 % | 15.4 % - 36.4 % | 28.1 % - 67.6 % | 4.33 % - 11.9 % |
| 30-54h | 20 % | 7.27 % - 18.5 % | 15.2 % - 36.4 % | 27.6 % - 65.9 % | 3.86 % - 10.7 % |
| 30-72h | 20 % | 5.97 % - 16.2 % | 15.1 % - 36.0 % | 24.6 % - 62.0 % | 3.50 % - 9.17 % |
| 48-54h | 20 % | 7.15 % - 17.8 % | 19.2 % - 42.8 % | 32.1 % - 71.2 % | 3.02 % - 9.38 % |
| 48-72h | 20 % | 5.89 % - 15.5 % | 19.4 % - 41.9 % | 30.1 % - 72.6 % | 2.95 % - 8.20 % |
| 54-72h | 20 % | 5.99 % - 16.0 % | 19.9 % - 45.5 % | 31.2 % - 74.6 % | 2.65 % - 8.13 % |
| 3-24h | 20 % | 9.30 % - 23.9 % | 9.16 % - 21.9 % | 15.2 % - 35.5 % | 8.73 % - 21.2 % |
| 3-30h | 20 % | 7.39 % - 19.5 % | 8.98 % - 22.2 % | 14.9 % - 34.9 % | 6.68 % - 17.7 % |
| 3-48h | 20 % | 4.97 % - 13.3 % | 9.58 % - 22.8 % | 14.5 % - 34.0 % | 4.69 % - 12.2 % |
| 3-54h | 20 % | 4.98 % - 13.4 % | 10.5 % - 23.0 % | 14.7 % - 34.3 % | 4.49 % - 11.6 % |
| 3-72h | 20 % | 4.76 % - 15.8 % | 10.6 % - 25.5 % | 14.6 % - 34.5 % | 4.45 % - 16.3 % |

TAT2: time spent above 0.02 IU/mL threshold, CL: clearance, V1: central volume, CV: coefficient of variation, pre: predose


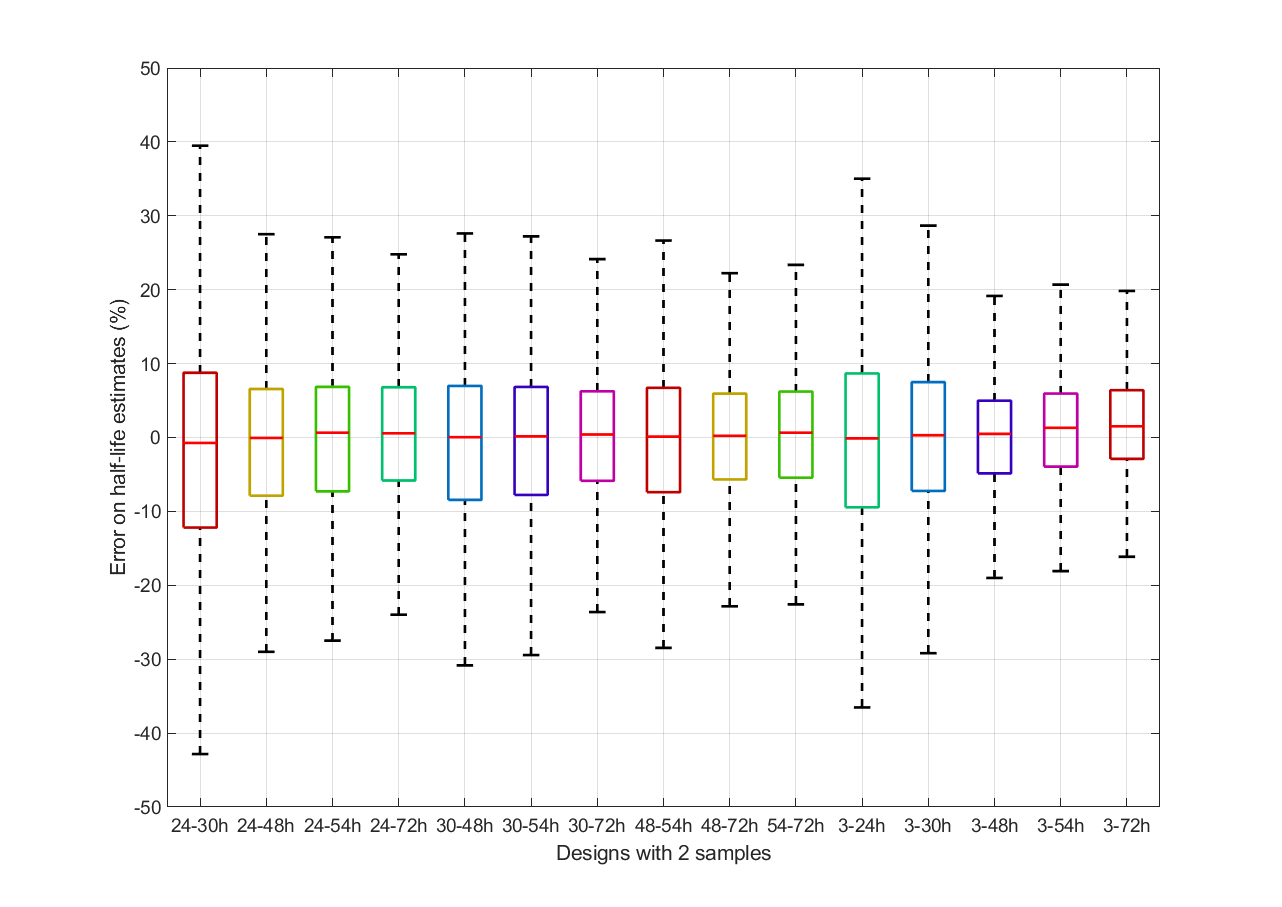


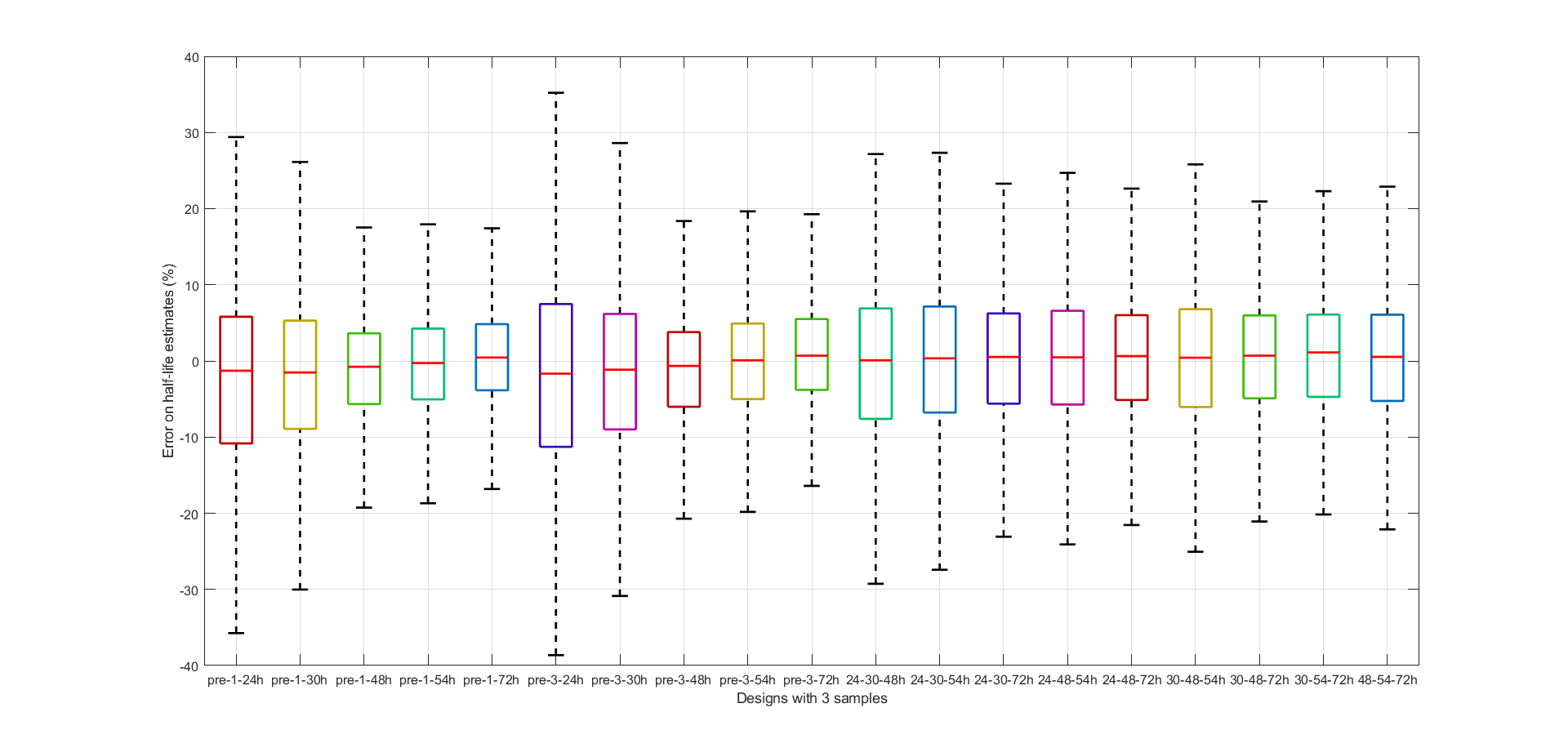


Figure S6: Limited analysis sampling results: boxplot of the error on half-life predictions for each 2- and 3- samples designs tested.
